# *Limosilactobacillus reuteri* DSM 17938 Inhibition of Biofilm Formation by *Prevotella intermedia* and *Fusobacterium nucleatum* Across Salivary pH: An In Vitro Study

**DOI:** 10.1055/s-0044-1786846

**Published:** 2024-06-28

**Authors:** Nissia Ananda, Vera Julia, Endang Winiati Bachtiar

**Affiliations:** 1 Faculty of Dentistry, Universitas Indonesia, Jakarta, Indonesia; 2 Staff of Dental Department, Universitas Indonesia Hospital, Jl. Prof Dr. Bahder Djohan, Universitas Indonesia, Pondok Cina, Beji, Depok, West Java, Indonesia; 3 Department of Oral and Maxillofacial Surgery, Faculty of Dentistry, Universitas Indonesia, Jakarta, Indonesia; 4 Department of Oral Biology, Faculty of Dentistry, Universitas Indonesia, Jakarta, Indonesia

**Keywords:** *Limosilactobacillus reuteri*, biofilm formation, salivary pH, *Prevotella intermedia*, *Fusobacterium nucleatum*, alveolar osteitis

## Abstract

**Objectives** This study aims to investigate *Limosilactobacillus reuteri* DSM 17938's antibiofilm effects on *Prevotella intermedia* and *Fusobacterium nucleatum* , common causes of alveolar osteitis. It seeks topical alternatives to prevent this condition posttooth extraction. The secondary objective is to assess these effects under different pH conditions (pH 4.5 and pH 7), mimicking oral cavity saliva pH dynamics.

**Materials and Methods** Ethical approval was secured for the saliva collection process involving five healthy adult participants who had undergone wisdom tooth extraction. Saliva samples were diligently collected on the 7th day post-surgery. The unstimulated saliva underwent a series of treatments, including the addition of phenylmethylsulfonyl fluoride (PMSF), pH adjustments, centrifugation, and filtration. The pH levels were re-measured, and subsequent adjustments were made to achieve pH values of 4.5 or 7. *Limosilactobacillus reuteri* DSM 17938, with a concentration of 1×10 ^8^ colony-forming units (CFU) per 5 drops, was utilized in the study. Biofilm testing involved incubating saliva samples with varying pH (4.5 or 7) alongside bacterial suspensions ( *Prevotella intermedia, Fusobacterium nucleatum* , or a mixed species). The Interlac suspension was introduced, and plates were anaerobically incubated for 24 hours. Biofilm results were obtained using a spectrometer. The test is conducted in triplicate.

**Statistical Analysis** To scrutinize the impact of pH on biofilm development, the acquired data underwent a two-way ANOVA test in SPSS as part of the statistical analysis. A significance level of *p* <0.05 was used to determine statistical significance.

**Results** *Limosilactobacillus reuteri* DSM 17938 significantly reduced biofilm formation across bacterial strains ( *p* = 0.000). Statistical analysis indicated a significant impact of pH on biofilm development ( *p* = 0.000) compared to no saliva samples, with higher formation observed under acidic conditions (pH 4.5). However, the pH levels of 4.5 and 7 did not result in significantly different bacterial biofilm formation ( *p* = 0.529).

**Conclusion** This research highlights *Limosilactobacillus reuteri* DSM 17938's potency in inhibiting biofilm formation of *Prevotella intermedia* and *Fusobacterium nucleatum* . Salivary pH variations significantly influence biofilm development, emphasizing the need to consider pH when assessing probiotic effectiveness. Despite limitations in saliva sample sterilization, this study provides valuable insights into alternative approaches for preventing alveolar osteitis. Further research should explore clinical applications and refine sterilization methods for more accurate results.

## Introduction

Alveolar osteitis, a frequently encountered complication that arises subsequent to tooth extraction, is characterized by a range of distressing symptoms. 1 2 3 Patients commonly experience intense and pulsating pain, accompanied by the presence of an unpleasant mouth odor, further complicating the act of eating. Despite the prevalence of this condition, the exact underlying causes remain shrouded in uncertainty. 1 4 5 However, several risk factors have been identified, considerably augmenting the likelihood of its occurrence. Notably, individuals with suboptimal oral hygiene practices and those affected by preexisting localized infections, such as pericoronitis or periodontitis, are at a significantly heightened risk of developing alveolar osteitis. In the context of this condition, it is noteworthy that both *Prevotella* and *Fusobacterium* , being fibrinolytic microorganisms, may induce blood clot lysis in the dental alveolus, potentially contributing to alveolar osteitis. They emerge as prominent bacterial players, adding a layer of complexity to the disease's etiology. 1 6 7

Exploring the intriguing domain of oral health, past investigations have unveiled the potential of probiotics to exert a positive influence on the intricate oral flora. This phenomenon is facilitated through bacteriotherapy mechanisms, which involve the stimulation of beneficial microflora while simultaneously inhibiting the proliferation of pathogenic bacteria. Probiotics, constituting a collection of beneficial live bacteria when administered in precise quantities, carry the promise of bestowing a range of health advantages. 8 9 10

*Limosilactobacillus reuteri* , among several studied probiotics, stands out for its antibacterial and immuno-inflammatory properties, effectively inhibiting the growth of pathogenic microorganisms. Multiple studies have highlighted its favorable impact on oral health. Furthermore, *Limosilactobacillus reuteri* demonstrates resilience against low pH conditions, although a healthy individual typically maintains salivary pH within the range of 6.2 to 7.6. 11 12 13 Salivary pH fluctuations can occur due to factors such as acidic food and drink consumption, gastroesophageal reflux disease, salivary gland dysfunction, chlorhexidine mouthrinse, and medication use. Typically, these variations return to their baseline levels within approximately 30 minutes, owing to the inherent buffering mechanisms in the human body. 12 Prior research found that salivary pH dropped to an average of 4.5 during the initial 0 to 10 minutes when lollipops were held in the buccal pouch, but subsequently reverted to the pH range of 6 to 7 after 15 minutes. 14

Given *Limosilactobacillus reuteri* 's resilience to low pH and the potentially acidic nature of saliva, this study's primary objective is to investigate its antibiofilm effects on *Prevotella intermedia* and *Fusobacterium nucleatum* , common causes of alveolar osteitis. This research aims to identify alternative topical agents for preventing alveolar osteitis posttooth extraction. The secondary objective is to assess these effects under different pH conditions (pH 4.5 and 7), reflecting the dynamic pH environment of the oral cavity.

## Materials and Methods

### Subject Selection

Ethical approval was obtained from The Dental Research Ethics Committee, Faculty of Dentistry, Universitas Indonesia (protocol number: 070520623); and The Ethics Committee of University of Indonesia Hospital (RSUI; protocol number: 2023-07-260), for saliva collection. The samples were collected from five donors on the 7th day following the surgery. Participant selection involved securing their voluntary participation by completing informed consent forms provided to them.

### Inclusion and Exclusion Criteria

The inclusion criteria encompassed healthy adult participants aged between 18 and 65 years old, with no systemic diseases, undergoing wisdom tooth extraction procedures at either the RSUI or the Special Hospital for Dental and Oral Health, Faculty of Dentistry, University of Indonesia (RSKGM FKG UI). Participants willingly consented to participate in the research by signing informed consent forms. Exclusion criteria comprised participants outside the 18 to 65 years age range, those with systemic diseases, and individuals unwilling to participate in the research.

### Saliva Collection

Unstimulated saliva samples are obtained using the spitting method and collected into sterile tubes with the addition of phenylmethylsulfonyl fluoride, with a volume of at least 2 to 4 mL collected. Subsequently, the pH of the saliva samples is measured using universal indicator pH paper, and the results are documented. The tubes are subsequently placed in a cooler box, kept chilled in an ice bath, and then stored in the laboratory refrigerator (frozen at −20°C) until the next stage of the research is conducted.

### Saliva Preparation

In the laboratory, the collected saliva underwent centrifugation (10 minutes, 4°C, 3,800 rpm), and the resulting supernatant was filtered using a filter membrane with a pore size of 0.22 μm. Subsequently, the pH of the filtered saliva was remeasured. After this, pH adjustments were carried out using either NaOH or HCl until reaching a pH of 4.5 or 7, respectively, preparing it for utilization in the subsequent stage of research.

### *Limosilactobacillus reuteri*

The probiotic employed was a suspension of *Limosilactobacillus reuteri* DSM 17938 (Interlac). The suspension included freeze-dried *Limosilactobacillus reuteri* DSM 17938, with a concentration of 1 × 10 ^8^ colony-forming units (CFU) per 5 drops.

### Biofilm Testing

Saliva with a pH of 4.5 or 7, which had been previously prepared, was dispensed in a volume of 50 microliters into the designated wells. Subsequently, the plates were incubated at 37°C for 30 minutes. After the incubation period, the saliva was carefully pipetted from the wells and discarded. Next, 100 microliters of the bacterial suspensions to be tested, including monospecies of *Prevotella intermedia* , monospecies of *Fusobacterium nucleatum* , or a mixed species of *Prevotella intermedia* and *Fusobacterium nucleatum* , were added to the wells. Following the addition of the bacterial suspensions, 100 microliters of the Interlac suspension was introduced into each well. The plates were then incubated under anaerobic conditions for 24 hours, utilizing an AnaeroPack within an air-tight container. The biofilm test results were subsequently read using a spectrometer at a wavelength of 600 nm to determine their optical density. The test was conducted in triplicate.

### Statistical Analysis

The acquired data underwent a distribution test. When the data demonstrated a normal distribution pattern, a two-way analysis of variance (ANOVA) analysis was applied. This approach allowed for the appropriate statistical analysis based on the underlying distribution of the data. The software used for statistical analysis is SPSS version 24. A significance level of *p* < 0.05 was used to determine statistical significance.

## Results

In this particular study, a total of five donors participated, with two donors recruited from the RSKGM FKG UI and three donors from the RSUI. Before the collection of saliva samples, each donor adhered to a protocol that involved rinsing their mouths with water and refraining from eating or drinking for a minimum of 2 hours. Subsequent analysis revealed that two donors exhibited a measured saliva pH of 6, whereas the remaining three donors had a saliva pH of 7, as determined using pH test strips.

The study's outcomes revealed distinct patterns in biofilm formation across various pH conditions and bacterial species in both treated and control groups ( Table 1 ). In the treated group receiving *Limosilactobacillus reuteri* DSM 17938, *Prevotella intermedia* monospecies exhibited its highest biofilm formation at pH 4.5, contrasting with its lowest formation at pH 7. Meanwhile, the control group lacking active ingredients showcased the highest biofilm formation for *Prevotella intermedia* monospecies in the absence of a saliva sample, with the lowest observed at pH 7. For *Fusobacterium nucleatum* monospecies within the treated group, the highest biofilm formation emerged at pH 7, while the lowest was detected in the absence of a saliva sample. Conversely, in the control group, the highest biofilm formation for *Fusobacterium nucleatum* monospecies was observed without a saliva sample, contrasting with its lowest formation at pH 7. In the case of the mixed species ( *Fusobacterium nucleatum and Prevotella intermedia* ), the treated group exhibited the highest biofilm formation without a saliva sample and the lowest at pH 7. Similarly, in the control group, the highest biofilm formation for the mixed species occurred without a saliva sample, while the lowest was at pH 4.5. These findings underscore variable biofilm formation patterns across different pH conditions and bacterial species in both treated and control groups.

**Table 1:** Optical density results of biofilm testing in treated and control groups with varied saliva pH

	*Limosilactobacillus reuteri* group			Control group		
	pH 4.5	pH 7	No saliva	pH 4.5	pH 7	No saliva
*Prevotella intermedia*	0.215 ± 0.119	0.197 ± 0.117	0.202 ± 0.002	0.267 ± 0.047	0.204 ± 0.018	0.356 ± 0.036
*Fusobacterium nucleatum*	0.201 ± 0.182	0.217 ± 0.010	0.192 ± 0.008	0.212 ± 0.013	0.194 ± 0.003	0.321 ± 0.107
Mixed	0.218 ± 0.014	0.209 ± 0.006	0.240 ± 0.008	0.241 ± 0.003	0.250 ± 0.022	0.305 ± 0.005

The analysis of the result data revealed a statistically normal distribution, as confirmed by the median indicator. Leveraging this normal distribution, a two-way ANOVA test was conducted using SPSS to further scrutinize and interpret the dataset. This statistical approach was employed to delve deeper into the relationship between multiple variables and their impact on the observed biofilm formation.

The two-way ANOVA analysis regarding biofilm formation's independent variable presented noteworthy outcomes ( Table 2 ). Salivary pH notably impacted biofilm development, with higher formation observed under acidic conditions (pH 4.5) compared to neutral saliva (pH 7). This trend remained consistent across *Prevotella intermedia* monospecies, *Fusobacterium nucleatum* monospecies, and the mixed species of *Prevotella intermedia* and *Fusobacterium nucleatum* . To obtain more detailed information about these results, we conducted a post hoc Bonferroni test.

**Table 2:** Results of two-way ANOVA test with biofilm formation as the dependent variable

Source	F	Sig.
PH	17.312	0.000 **
Interlac	37.459	0.000 **
Bacteria	2.398	0.105
PH * Interlac	15.583	0.000 **
PH * bacteria	0.994	0.423
Interlac * bacteria	1.453	0.247
PH * Interlac * bacteria	2.PM: 079	0.104

Abbreviation: ANOVA, analysis of variance

* The statistical analysis performed to check the relation between variables stated.

** *p* < 0.05 was considered to be statistically significant.

The outcomes of the post hoc Bonferroni tests revealed significant differences in bacterial biofilm formation between saliva with a pH of 4.5 and samples without saliva ( table 3 ). This suggests that the acidity level at pH 4.5 has a distinct impact on the formation of bacterial biofilms compared to the absence of saliva. Similarly, the post hoc Bonferroni analysis demonstrated significant differences in bacterial biofilm formation between saliva with a pH of 7 and samples without saliva. This indicates that the neutral pH of 7 also plays a role in influencing the formation of bacterial biofilms when compared to the absence of saliva. However, interestingly, the results of the post hoc Bonferroni test indicated no significant difference in bacterial biofilm formation between saliva with a pH of 4.5 and saliva with a pH of 7. This suggests that within the scope of this study, the pH levels of 4.5 and 7 do not lead to significantly different bacterial biofilm formation.

**Table 3:** Results of the post hoc Bonferroni test comparing the biofilm formation in saliva at different pH levels

Level of pH		Sig.
PH 4.5	pH 7	0.529
	No saliva	0.000 *
pH 7	No saliva	0.000 *

* *p* < 0.05 was considered to be statistically significant.

In addition, based on the two-way ANOVA test, the application of *Limosilactobacillus reuteri* DSM 17938 significantly reduced biofilm formation, proving effective across *Prevotella intermedia* , *Fusobacterium nucleatum* , and the mixed species. Furthermore, there were no significant variations in biofilm formation among different bacterial types ( *Prevotella intermedia* , *Fusobacterium nucleatum* , or mixed species), suggesting a consistent quantity of biofilm regardless of the bacterial strain. Lastly, the combined use of *Limosilactobacillus reuteri* DSM 17938 and controlled salivary pH exhibited a substantial impact on biofilm formation. This underscores the significant influence of these factors on biofilm development across various bacterial species investigated in this study.

## Discussion

Lately, there has been a growing interest in enhancing a healthy oral microbiome and preventing diseases by utilizing probiotic bacteria. 15 In general, several *lactobacilli* strains have been proposed as adjuncts to “good clinical practice” for managing various oral health issues such as childhood caries, gingivitis, periodontal disease, candidiasis, and halitosis. Furthermore, these beneficial bacteria might have the potential in enhancing the healing process of surgical wounds by influencing the immune response systemically and through the up-regulation of the neuropeptide hormone oxytocin. 16 However, the application of probiotics, especially *Limosilactobacillus reuteri* , within the field of oral and maxillofacial surgery remains limited. There are only a few articles connecting *Limosilactobacillus reuteri* to oral maxillofacial surgery, predominantly focusing on its effects in scenarios like third molar extraction and wound healing. 16 17 This study aims to contribute insights into the utilization of *Limosilactobacillus reuteri* in oral maxillofacial surgery, despite the fact that the research was conducted in an in vitro setting. The findings seek to expand understanding regarding the potential benefits of *Limosilactobacillus reuteri* application within this surgical context.

Dry socket represents one of the most distressing complications following tooth extraction. Its management poses a considerable challenge, especially when conventional treatments prove ineffective. 2 The preemptive use of antibiotics, such as penicillins or nitroimidazoles, has demonstrated a significant reduction in the occurrence of dry socket and/or infection subsequent to third molar extraction. 18 This may be connected to microorganisms such as *Prevotella* and *Fusobacterium* contributing to alveolar osteitis. 1 6 Yet, the utilization of antibiotics may contribute to the emergence of antibiotic resistance, which is currently recognized as a global public health emergency by the World Health Organization, termed a silent pandemic. 19 Hence, it becomes imperative to explore alternative antimicrobial options beyond antibiotics that can effectively reduce the risk of alveolar osteitis posttooth extraction.

*Limosilactobacillus reuteri* exhibits a multifaceted approach in combating oral issues. It showcases antibacterial properties, actively restraining the growth of harmful microorganisms. Additionally, its immunoinflammatory characteristics modulate the immune response in the oral cavity, contributing to its therapeutic potential against a range of oral conditions. 11 12 13 Notably, *Limosilactobacillus reuteri* strains demonstrate remarkable antiplaque effects by hindering microorganism adhesion and growth on tooth surfaces. This bacterium alters dental plaque biochemistry, lessening cytotoxic product production and obstructs the formation of intercellular plaque matrices. 20 These collective actions highlight the comprehensive role of *Limosilactobacillus reuteri* in combating plaque formation, essential in preventing various oral pathologies.

Exploring alternative drug options demands a comprehensive understanding of various considerations. One crucial factor involves ensuring the stability of these alternatives across diverse oral conditions. For instance, the viability of probiotics can be influenced by multiple factors, with the acidic condition being one of the reported elements affecting their effectiveness. Generally, a healthy individual maintains a salivary pH within the range of 6.2 to 7.6. 11 12 13 Nevertheless, salivary pH can fluctuate due to various factors. 12 13 These fluctuations highlight the dynamic nature of oral pH regulation and its potential influence on the effectiveness of antimicrobial agents. 14 This emphasizes the importance of considering these pH changes when exploring alternative options for managing oral conditions such as alveolar osteitis.

This study delved into examining the antibiofilm effects of *Limosilactobacillus reuteri* DSM 17938 on *Prevotella intermedia* and *Fusobacterium nucleatum* . It thoroughly investigated these effects within both monospecies and mixed-species biofilms, scrutinizing diverse saliva pH conditions at pH 4.5 and 7. By encompassing both acidic and normal pH ranges, this research provided a comprehensive understanding of *Limosilactobacillus reuteri* 's impact on biofilms. Surprisingly, the study unveiled that the acidic state of saliva with a pH of 4.5 exhibited no significant variance in inhibiting biofilm formation compared to saliva with a pH of 7 regarding *Limosilactobacillus reuteri* 's effects on these biofilms. Nevertheless, notable discrepancies in bacterial biofilm formation were noted between saliva samples with pH levels of 4.5 and 7, distinctly different from samples lacking saliva. This groundbreaking exploration into *Limosilactobacillus reuteri* 's antibiofilm effect under diverse saliva pH conditions underscores the necessity of considering salivary variables. These discoveries illuminate possible explanations for the varying results noticed in prior in vitro and clinical research concerning the antibiofilm impact of *Lactobacillus reuteri* . They underscore the significance of contextual elements like saliva pH when assessing its effectiveness in handling oral conditions such as alveolar osteitis.

The results of this research indicate that the application of *Limosilactobacillus reuteri* inhibits the formation of biofilm in monospecies of *Prevotella intermedia* , monospecies of *Fusobacterium nucleatum* , and the mixed species of *Prevotella intermedia* and *Fusobacterium nucleatum* . This inhibition might be attributed to the antibacterial capability of *Limosilactobacillus reuteri* . However, it's worth noting that previous clinical studies have yielded controversial outcomes regarding the antimicrobial effects of *Limosilactobacillus reuteri* on *Prevotella intermedia* and *Fusobacterium nucleatum* . This discrepancy in findings could be influenced by variations in sample collection methods, with some studies using plaque samples while others used saliva. Additionally, prior research has not consistently considered pH as a variable of interest. 21 However, this study revealed that differences in salivary pH could indeed influence biofilm formation.

The research outcomes underscore the potency of *Limosilactobacillus reuteri* in restraining biofilm formation across various bacterial strains. Notably, the application of *Limosilactobacillus reuteri* exhibited a notable inhibitory effect on biofilm formation in *Prevotella intermedia* monospecies, *Fusobacterium nucleatum* monospecies, and the mixed species of *Prevotella intermedia* and *Fusobacterium nucleatum* . This observed inhibition might be attributed to the documented antibacterial potential of *Limosilactobacillus reuteri* . Previous clinical studies present divergent findings regarding its antimicrobial effects on *Prevotella intermedia* and *Fusobacterium nucleatum* . 21 For instance, Iniesta et al, conducting research in Spain on gingivitis patients, noted that *Limosilactobacillus reuteri* application for 28 days effectively suppressed the overall count of pathogenic bacteria, including *Prevotella intermedia* and *Fusobacterium nucleatum* , in saliva samples. 22 Similarly, Vivekananda et al in India, studying chronic periodontitis patients, found that *Limosilactobacillus reuteri* tablets administered for 21 days significantly reduced the count of *Prevotella intermedia* bacteria. 23 Contrastingly, Laleman et al in Belgium, focusing on subjects with peri-implantitis, discovered that administering *Limosilactobacillus reuteri* drops for 12 weeks notably suppressed the count of *Prevotella Intermedia* in subgingival and tongue samples. However, they observed no significant differences in saliva samples over the 24-week observation period. 24

Moreover, Tada et al in Japan investigated peri-implantitis patients and found that *Limosilactobacillus reuteri* tablet usage for 24 weeks did not significantly affect the count of *Prevotella intermedia* and *Fusobacterium nucleatum* . However, they highlighted that patients were administered azithromycin antibiotics for the initial 3 days, potentially impacting the research outcomes. 25 Furthermore, diverse studies by Hallström et al in Sweden, Peña et al, and Galofré et al in Spain, examining peri-implant mucositis and peri-implantitis patients, showed varied outcomes concerning the antibacterial effects of *Limosilactobacillus reuteri* on *Prevotella intermedia* and *Fusobacterium nucleatum* . 26 27 28 These research disparities underscore the complexity of microbial interactions within oral ecosystems and emphasize the multifaceted influences contributing to divergent research outcomes. These influential factors encompass variations in study designs, patient cohorts with distinct oral health conditions, diverse durations of probiotic interventions, and the exclusion of pH as a pivotal variable.

One limitation of this study lies in the sterilization process applied to the collected saliva samples using filtration methods. Filtration, while widely adopted and preferred, has been associated with potential drawbacks. It has been reported that filtration methods may lead to a reduction in the total amount of salivary proteins and enzyme activities. 29 Nevertheless, the use of natural saliva that has been filtered is still preferred over artificial saliva in order to simulate oral cavity conditions in in vitro studies.

In conclusion, *Limosilactobacillus reuteri* DSM 17938 has demonstrated an inhibitory capability against the biofilm formation of *Prevotella intermedia* and *Fusobacterium nucleatum* , whether in monospecies or mixed species. Moreover, the performance of *Limosilactobacillus reuteri* DSM 17938 in inhibiting biofilm formation was notably enhanced under the condition of saliva of pH 7 compared to pH 4.5. Both the administration of *Limosilactobacillus reuteri* DSM 17938 and salivary acidity levels significantly influenced the formation of biofilms by *Prevotella intermedia* and *Fusobacterium nucleatum* , irrespective of their mono- or mixed-species nature. This study lays the groundwork for exploring alternatives in probiotics, aiming to prevent alveolar osteitis.
